# Cancer Special Issue: Early detection and minimal residual disease

**DOI:** 10.1371/journal.pmed.1003794

**Published:** 2021-10-12

**Authors:** Beryne Odeny

**Affiliations:** PLOS Medicine, San Francisco, California, United States of America

## Abstract

Beryne Odeny discusses PLOS Medicine’s Special Issue on early cancer detection and minimal residual disease.

PLOS Medicine’s editorial team, together with guest editors, Chris Abbosh, Sarah-Jane Dawson and Charles Swanton, are delighted to disseminate several high-quality translational research and clinical studies on advances in early cancer detection. In 2020 alone, there were upward of 19 million new cancer cases and 10 million cancer deaths, worldwide [1]. Cancer kills more people than HIV/AIDS, tuberculosis and malaria combined and should be a top health priority, regardless of region or country [2]. Early detection of cancer and identification of minimal residual disease (MRD), post-treatment, are key to timely treatment and cure. This issue features robust studies that bring cutting edge, and potentially scalable, innovations that have the potential to inform research, policy, and clinical cancer management.

Three studies in this issue center on innovations for detection of MRD. Yaqi Wang and colleagues found that combining circulating tumor DNA (ctDNA) and Magnetic Resonance Imaging (MRI) improved prediction of response to neoadjuvant chemoradiotherapy in patients with locally advanced rectal cancer (LARC) before surgery [3]. This combined model also improved stratification of patients at high risk of recurrence, and clearly has important clinical implications for management of LARC as it could potentially inform guidelines on patient selection for non-operative management and targeted treatment strategies for those with highly recurrent diseases. Jeanne Tie and colleagues confirmed the prognostic utility of post-surgery and post-chemotherapy ctDNA in determining the risk of relapse among patients with colorectal cancer with liver metastases (CRCLM) [4]. They demonstrated the function of serial ctDNA measurement as an early marker of treatment of efficacy. This is a noteworthy advance that requires further study around optimized integration of ctDNA analyses in adjuvant chemotherapy for resectable CRCLM. Pradeep S. Chauhan and colleagues applied next-generation sequencing (NGS) for urine tumor DNA (utDNA) detection to assess MRD in patients with muscle-invasive bladder cancer who received neoadjuvant chemotherapy [5]. They found that MRD detection prior to radical cystectomy correlated with pathologic response and may be used to identify candidates for bladder sparing treatment. Urine tumor DNA also offers the ability to determine tumor mutational burden and can therefore facilitate personalized immunotherapy.

Two studies in this issue focused on early cancer detection. Jeffrey J. Szymanski and colleagues investigated the use of plasma cell-free DNA (cfDNA) ultra-low-pass whole genome sequencing (ULP-WSG) to distinguish the malignant peripheral nerve sheath tumor (MPNST) from its benign precursor lesion–plexiform neurofibroma–in patients with Neurofibromatosis type1(NF1) [6]. This provides a strong evidence base for use of plasma cfDNA in liquid biopsy to distinguish early between benign and malignant tumors of this hereditary cancer. This is proof of concept that cfDNA can be leveraged as a biomarker for monitoring treatment response in patients with MPNST. Brian D Nicholson and colleagues demonstrated that risk scores based on combinations of risk factors and routine blood tests can be used to stratify patients with unexpected weight loss based on their risk of cancer [7]. They found that these combined risk scores showed superior clinical utility–compared to the symptoms-only model–to discriminate between patients with and without cancer. In this, they clearly demonstrate innovation in the use of routine clinical tools at scale. This type of model could potentially be scaled-up in under-resourced settings.

With growing global interest in cancer diagnostics and treatment, these robust assays and tools are a welcome addition to the early cancer detection armamentarium, prior to and post-treatment. Further innovation around low-cost technologies and tools for early detection that can be rapidly tested and scaled up will further galvanize, the universal commitment to defeat cancer in both high and low resource settings.
